# Supplementary material for: Analysis of Culturable Bacterial Diversity of Pangong Tso Lake via a 16S rRNA Tag Sequencing Approach
Source: Microorganisms. 2024 Feb 17;12(2):397. doi: 10.3390/microorganisms12020397 (PMC10892101; doi:10.3390/microorganisms12020397)
Supplement: Supplementary file 1 [file microorganisms-12-00397-s001.zip › microorganisms-2772852-supplementary/supplementary figures.pptx]

## Slide 1
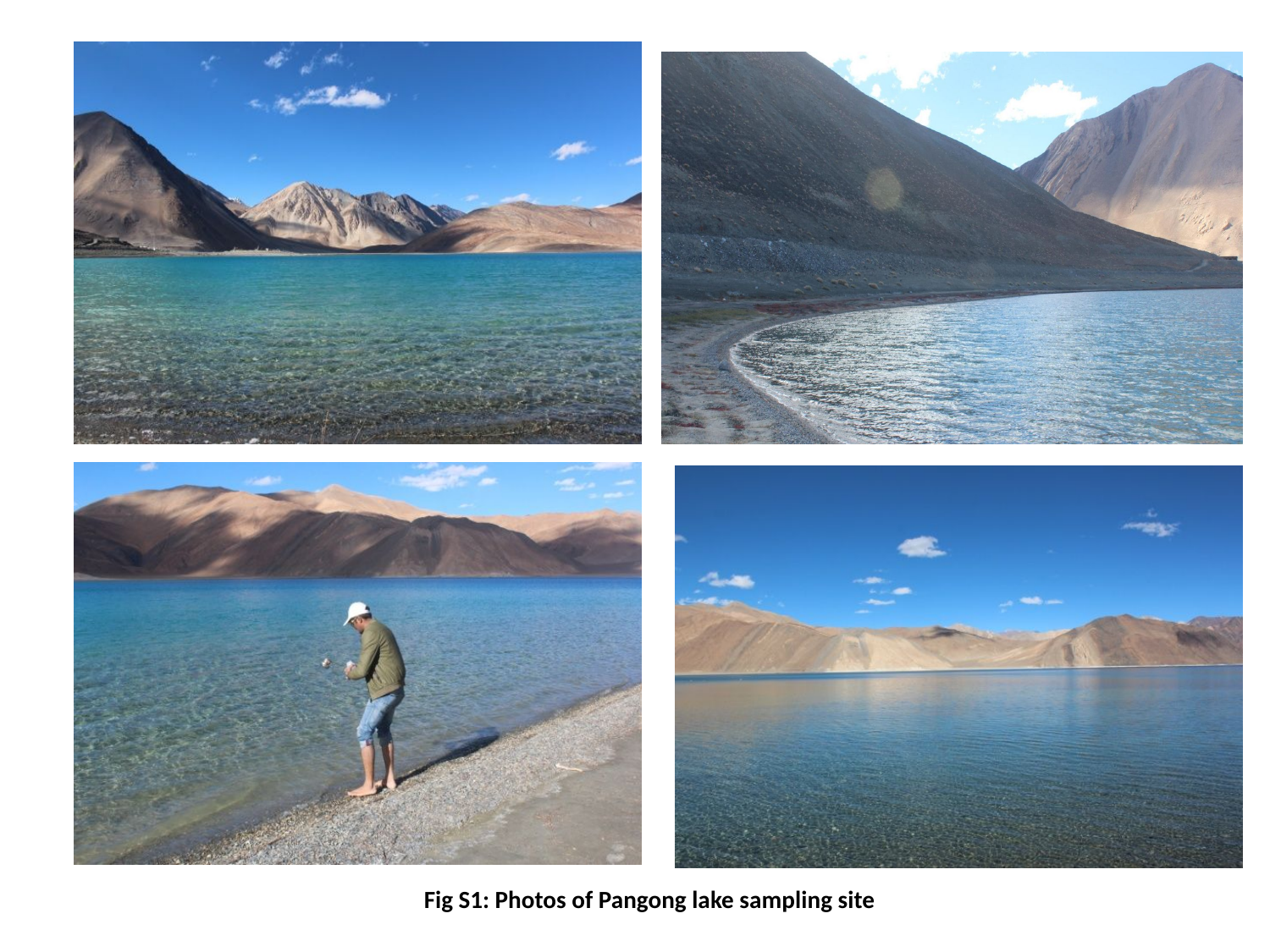

Fig S1: Photos of Pangong lake sampling site

## Slide 2
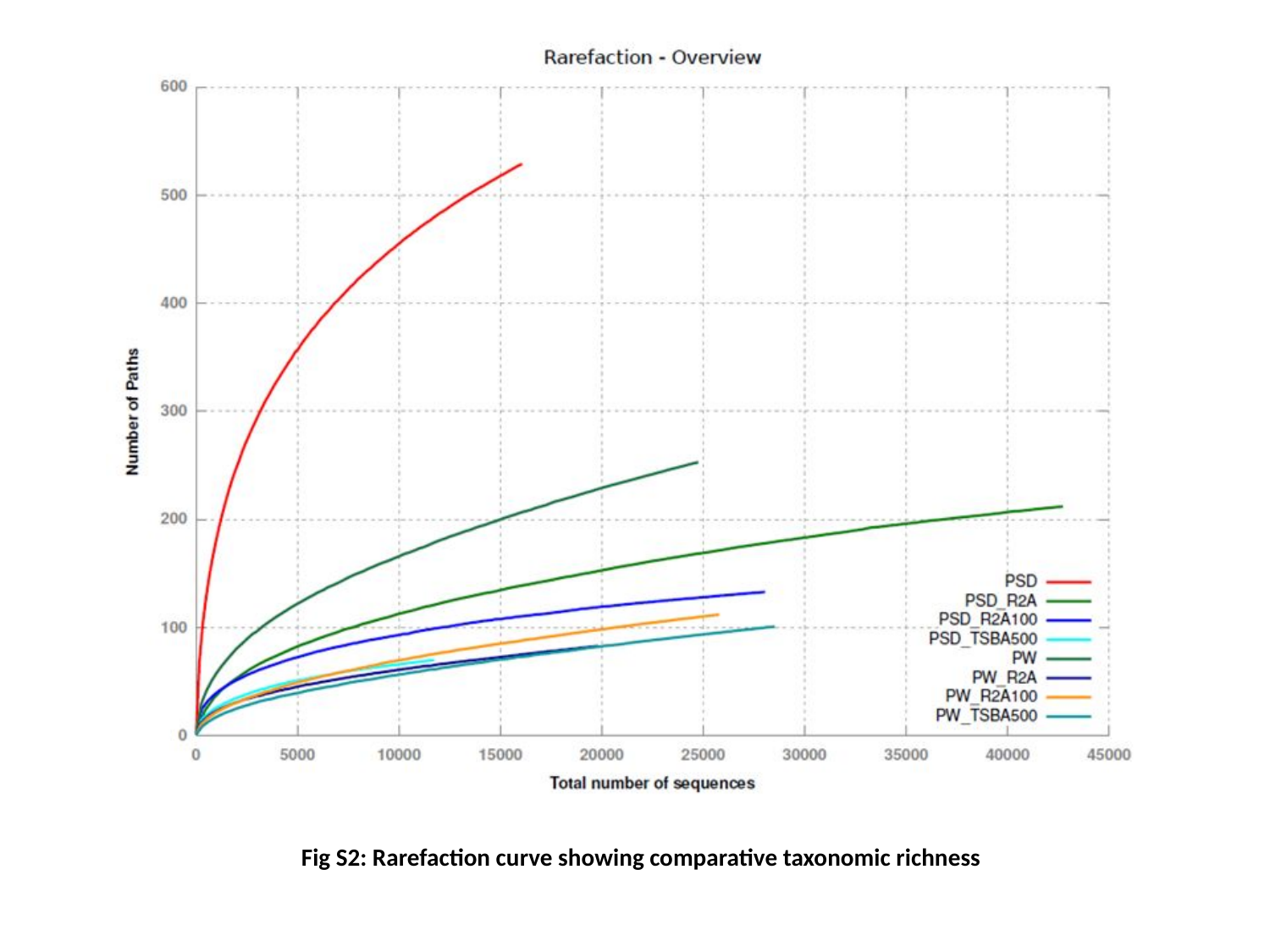

Fig S2: Rarefaction curve showing comparative taxonomic richness

## Slide 3
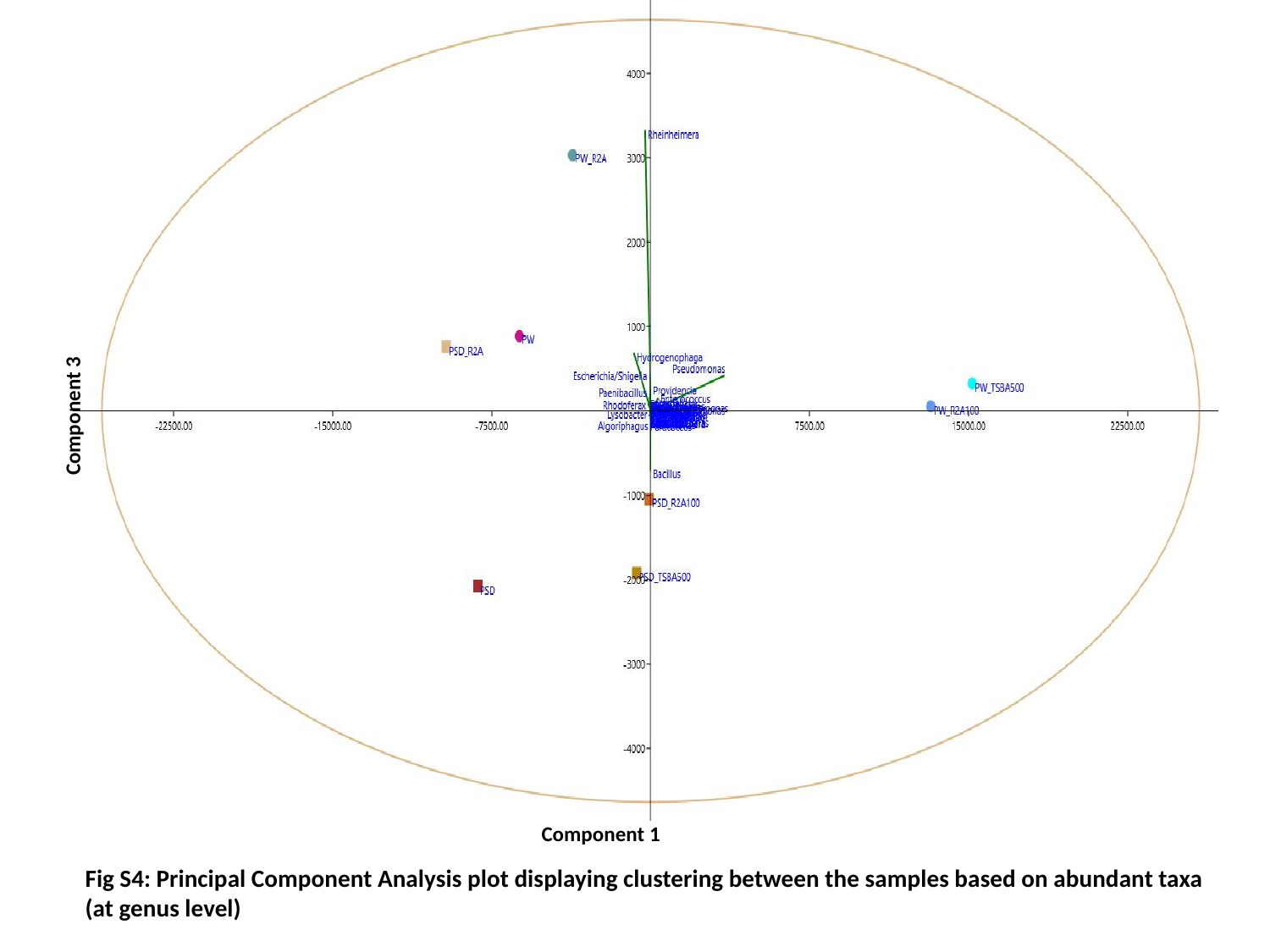

Component 3
Component 1
# Wo_BP
Fig S4: Principal Component Analysis plot displaying clustering between the samples based on abundant taxa (at genus level)
