# Supplementary material for: Analysis of Culturable Bacterial Diversity of Pangong Tso Lake via a 16S rRNA Tag Sequencing Approach
Source: Microorganisms. 2024 Feb 17;12(2):397. doi: 10.3390/microorganisms12020397 (PMC10892101; doi:10.3390/microorganisms12020397)
Supplement: Supplementary file 1 [file microorganisms-12-00397-s001.zip › microorganisms-2772852-supplementary/Table S1. Physicochemical.pdf]

Table S1. Physico-chemical analysis of Pangong sediment sample.

S.No.	Parameters	Pangong sediment	Unit
1	Conductivity of sediment$^{\alpha}$	1.565	mmho/cm
2	Total organic carbon (TOC)$^{\theta}$	0.122	%
3	Total kjeldahl nitrogen (TKN)*	0.46	mg/kg
4	Total Dissolved Solids (TDS)$^{\Psi}$	5450	mg/kg
5	Phosphorus$^{\Psi}$	11	mg/kg
6	Sulphate$^{\Psi}$	BDL	mg/kg
7	Ammonical nitrogen$^{\Psi}$	34	mg/kg
8	Nitrate nitrogen$^{\Psi}$	5	mg/kg
9	Nitrite (as NO2)$^{\Psi}$	0.20	mg/kg
10	Calcium (as Ca)$^{\omega}$	3889	mg/kg
11	Magnesium (as Mg)$^{\omega}$	1287	mg/kg
12	Zn (as Zn)$^{\omega}$	28.47	mg/kg
13	Manganese (as Mn)$^{\omega}$	548.37	mg/kg
14	Iron (as Fe)$^{\omega}$	1.47	%
15	Boron (as B)$^{\omega}$	71.90	mg/kg
16	Copper (as Cu)$^{\omega}$	46.66	mg/kg
17	Cobalt (as Cd)$^{\omega}$	9.5	mg/kg
18	Molybdenum (as Mo)$^{\omega}$	0.56	mg/kg
19	Cadmium (Cd)$^{\omega}$	0.12	mg/kg
20	Chromium (Cr)$^{\omega}$	17.28	mg/kg
21	Aluminium (as Al)$^{\omega}$	0.58	%
22	Barium $^{\omega}$	47.14	mg/kg
23	Nickel (as Ni)$^{\omega}$	12.98	mg/kg
24	Lead (as Pb)$^{\omega}$	5.84	mg/kg
25	Mercury (as Hg)$^{\omega}$	BDL	mg/kg
26	Arsenic (As)$^{\omega}$	15.09	mg/kg
27	Chloride (as Cl)$^{\Psi}$	520	mg/kg
28	Salinity$^{\alpha}$	0.74	ppt
29	Lithium$^{\omega}$	14.70	mg/kg
30	Vanadium$^{\omega}$	28.80	mg/kg
31	Selenium (as Se)$^{\omega}$	0.24	mg/kg
32	Silicon (Si)$^{\omega}$	400	mg/kg

$^{\alpha}$APHA2510 B, $^{\theta}$ IS 2720, *AOAC 955.04, $^{\Psi}$ Baruah & Barthakur, 1998, $^{\omega}$ AOAC 990.08.
