# Supplementary figures and images for: Analysis of Culturable Bacterial Diversity of Pangong Tso Lake via a 16S rRNA Tag Sequencing Approach
Source: Microorganisms. 2024 Feb 17;12(2):397. doi: 10.3390/microorganisms12020397 (PMC10892101; doi:10.3390/microorganisms12020397)

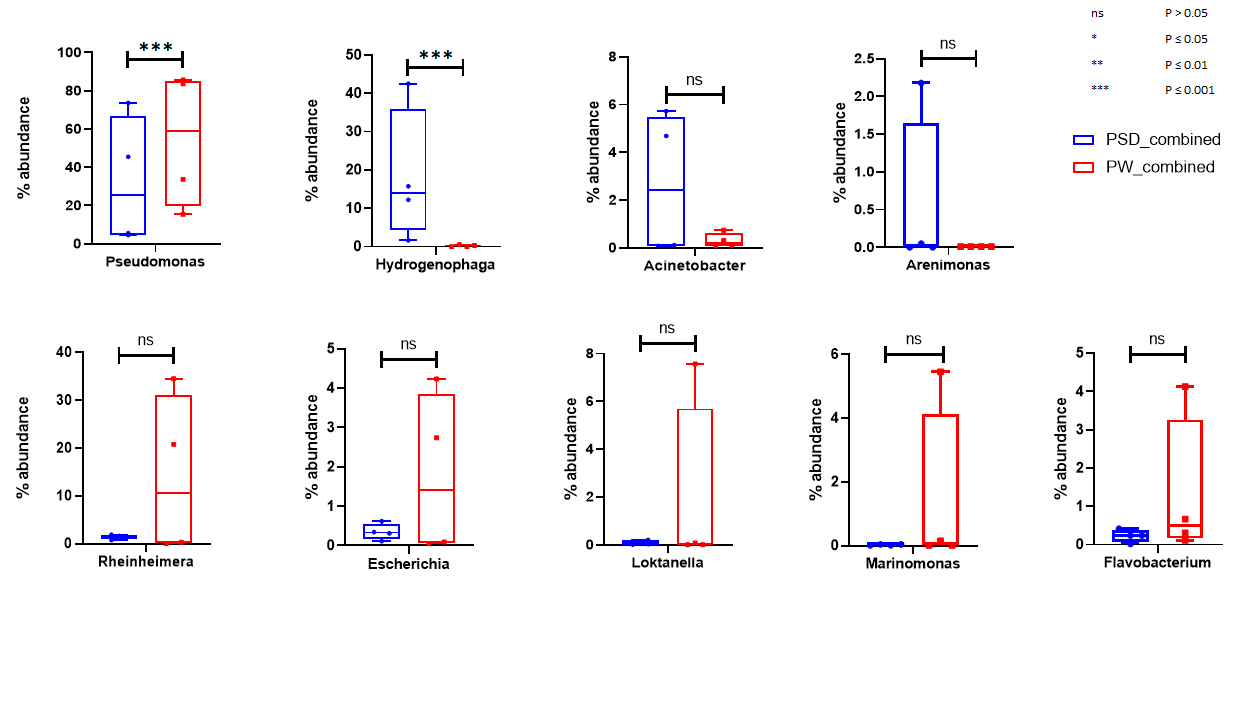

Supplement: Supplementary file 1 [file microorganisms-12-00397-s001.zip › microorganisms-2772852-supplementary/Figure S5.tif]

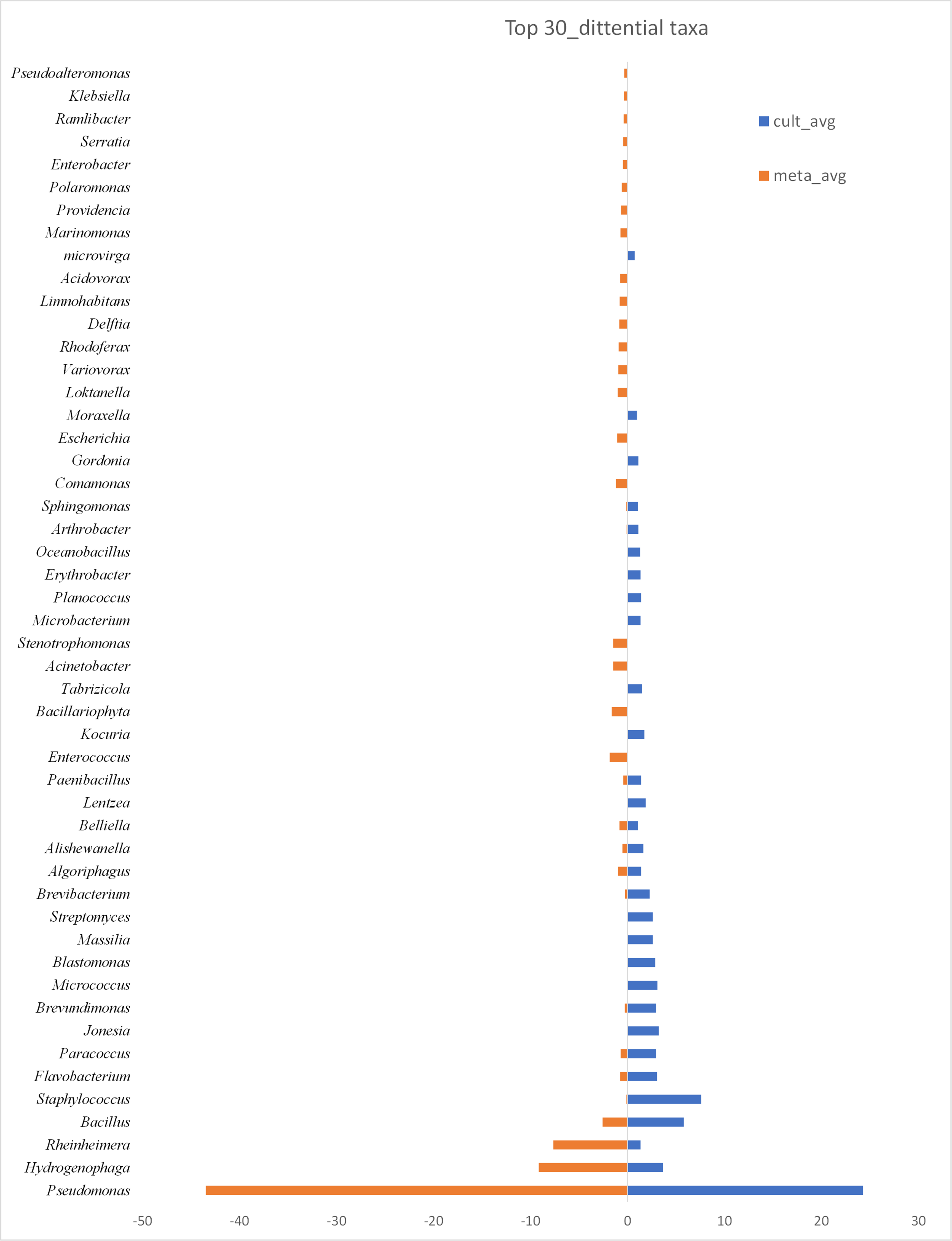

Supplement: Supplementary file 1 [file microorganisms-12-00397-s001.zip › microorganisms-2772852-supplementary/figure S6.tif]
