# Supplementary material for: Analysis of Culturable Bacterial Diversity of Pangong Tso Lake via a 16S rRNA Tag Sequencing Approach
Source: Microorganisms. 2024 Feb 17;12(2):397. doi: 10.3390/microorganisms12020397 (PMC10892101; doi:10.3390/microorganisms12020397)
Supplement: Supplementary file 1 [file Figure S3.pdf › microorganisms-2772852-supplementary/Figure S3.pdf]

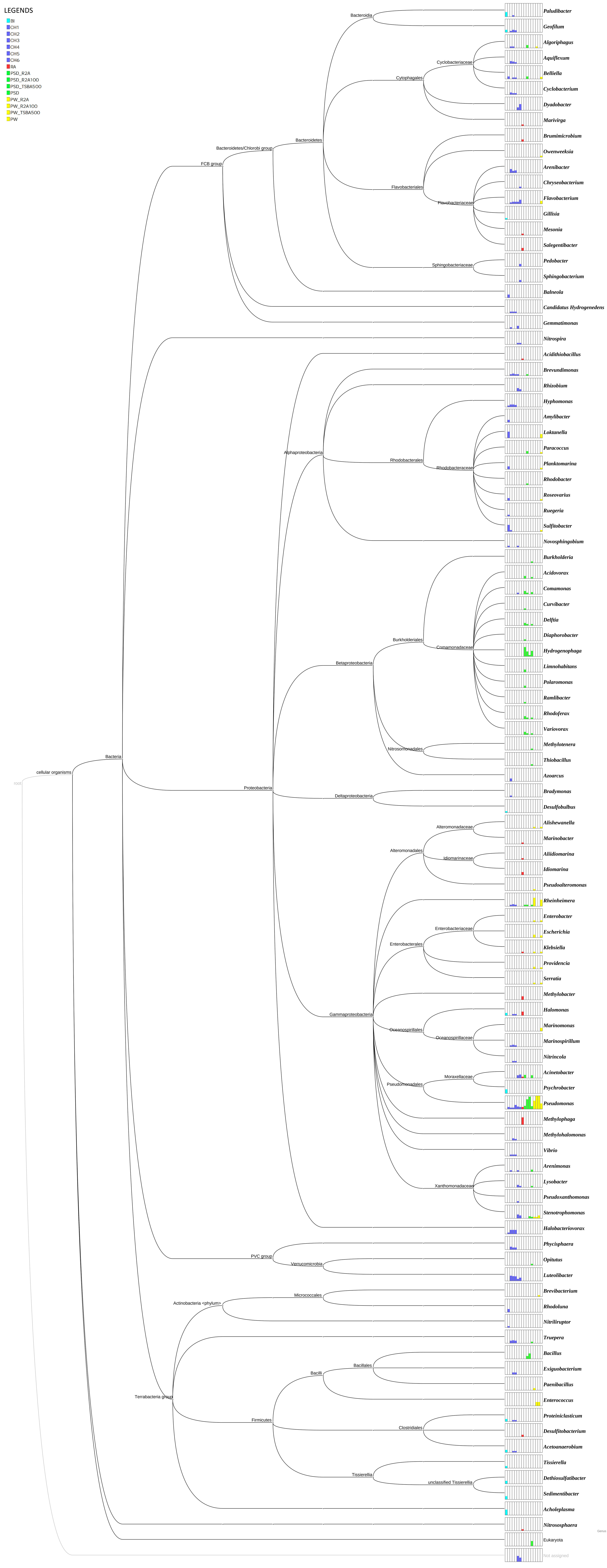
LEGENDS
BI
CH1
CH2
CH3
CH4
CH5
CH6
RA
PSD_R2A
PSD_R2A100
PSD_TSBA500
PSD
PW_R2A
PW_R2A100
PW_TSBA500
PW
root
cellular organisms
Bacteria
FCB group
Bacteroidetes/Chlorobi group
Bacteroidetes
Bacteroidia
Cytophagales
Cyclobacteriaceae
Flavobacteriales
Flavobacteriaceae
Sphingobacteriaceae
Paludibacter
Geofilum
Algoriphagus
Aquiflexum
Belliella
Cyclobacterium
Dyadobacter
Marivirga
Brumimicrobium
Owenweeksia
Arenibacter
Chryseobacterium
Flavobacterium
Gillisia
Mesonia
Salegentibacter
Pedobacter
Sphingobacterium
Balneola
Candidatus Hydrogenedens
Gemmatimonas
Nitrospira
Proteobacteria
Alphaproteobacteria
Rhodobacterales
Rhodobacteraceae
Acidithiobacillus
Brevundimonas
Rhizobium
Hyphomonas
Amylibacter
Loktanella
Paracoccus
Planktomarina
Rhodobacter
Roseovarius
Ruegeria
Sulfitobacter
Novosphingobium
Betaproteobacteria
Burkholderiales
Comamonadaceae
Nitrosomonadales
Burkholderia
Acidovorax
Comamonas
Curvibacter
Delftia
Diaphorobacter
Hydrogenophaga
Limnohabitans
Polaromonas
Ramlibacter
Rhodoferax
Variovorax
Methylotenera
Thiobacillus
Azoarcus
Deltaproteobacteria
Bradymonas
Desulfobulbus
Gammaproteobacteria
Alteromonadales
Alteromonadaceae
Idiomarinaceae
Enterobacterales
Enterobacteriaceae
Oceanospirillales
Oceanospirillaceae
Pseudomonadales
Moraxellaceae
Xanthomonadaceae
Alishewanella
Marinobacter
Aliidiomarina
Idiomarina
Pseudoalteromonas
Rheinheimera
Enterobacter
Escherichia
Klebsiella
Providencia
Serratia
Methylobacter
Halomonas
Marinomonas
Marinospirillum
Nitrincola
Acinetobacter
Psychrobacter
Pseudomonas
Methylophaga
Methylohalomonas
Vibrio
Arenimonas
Lysobacter
Pseudoxanthomonas
Stenotrophomonas
Halobacteriovorax
PVC group
Verrucomicrobia
Phycisphaera
Opitutus
Luteolibacter
Terrabacteria group
Actinobacteria <phylum>
Micrococcales
Brevibacterium
Rhodoluna
Nitriliruptor
Truepera
Firmicutes
Bacilli
Bacillales
Clostridiales
Tissierellia
unclassified Tissierellia
Bacillus
Exiguobacterium
Paenibacillus
Enterococcus
Proteiniclasticum
Desulfitobacterium
Acetoanaerobium
Tissierella
Dethiosulfatibacter
Sedimentibacter
Acholeplasma
Nitrososphaera
Eukaryota
Not assigned
Genus
